# Diversity of Toxigenic Fungi in Livestock and Poultry Feedstuffs

**DOI:** 10.3390/ijerph19127250

**Published:** 2022-06-13

**Authors:** Eman Khalifa, Marwa T. Mohesien, Monga I. Mossa, Magdalena Piekutowska, Amnah Mohammed Alsuhaibani, Basel A. Abdel-Wahab, Sotohy Ahmed Sotohy, Soumya Ghosh, Yosra A. Helmy, Mohamed Hussein, Ahmed M. Abdel-Azeem

**Affiliations:** 1 Department of Microbiology, Faculty of Veterinary Medicine, Matrouh University, Matrouh 51511, Egypt; khalifa.eman@alexu.edu.eg; 2 Botany and Microbiology Department, Faculty of Science, Damietta University, New Damietta 34511, Egypt; marwabotany@yahoo.com; 3 Botany and Microbiology Department, Faculty of Science, University of Al Arish, North Sinai 45516, Egypt; monga.ibrahim@aru.edu.eg; 4 Department of Geoecology and Geoinformation, Institute of Biology and Earth Sciences, Pomeranian University in Słupsk, Partyzantów 27, 76-200 Słupsk, Poland; magdalena.piekutowska@apsl.edu.pl; 5 Department of Physical Sport Science, College of Education, Princess Nourah bint Abdulrahman University, P.O. Box 84428, Riyadh 11671, Saudi Arabia; amalsuhaibani@pnu.edu.sa; 6 Department of Medical Pharmacology, College of Medicine, Assiut University, Assiut 7111, Egypt; basel_post@msn.com; 7 Department of Pharmacology, College of Pharmacy, Najran University, Najran P.O. Box 1988, Saudi Arabia; 8 Department of Zoonosis and Animal Health, Faculty of Veterinary Medicine, Assiut University, Assiut 71515, Egypt; sotohy2000@yahoo.com; 9 Department of Genetics, Faculty of Natural and Agricultural Sciences, University of the Free State, Bloemfontein 9301, South Africa; ghoshs@ufs.ac.za; 10 Department of Animal Hygiene, Zoonoses and Animal Ethology, Faculty of Veterinary Medicine, Suez Canal University, Ismailia 41522, Egypt; yosra_helmy@vet.suez.edu.eg; 11 Department of Veterinary Science, College of Agriculture, Food, and Environment, University of Kentucky, Lexington, KY 40503, USA; 12 Plant Pathology, Faculty of Agriculture and Natural Resources, Aswan University, Aswan 81528, Egypt; mmon3m@gmail.com; 13 Botany and Microbiology Department, Faculty of Science, University of Suez Canal, Ismailia 41522, Egypt

**Keywords:** animal feedstuff samples, fungi, *Aspergillus* species, aflatoxins, HPLC, mycotoxins

## Abstract

The purpose of this study was to discover how abundant toxigenic fungi and mycotoxins are in animal feedstuff samples. A total of ninety samples representing various types of animal feedstuff samples were collected from ninety sites in Egypt. Isolation, identification, and determination of mycotoxins (aflatoxins B1, B2, G1, G2, and ochratoxin A) were performed. The results revealed that 79 (87.77%) of the samples were contaminated with fungi, and 1.1 × 10^5^ CFU/g were recovered, including 41 fungal species belonging to 18 genera, such as Zygomycota, which was represented by three species (7.31% of the total species number), teleomorphic Ascomycota (10 species, 24.39%), and anamorphic Ascomycota (28 species, 69.29%). When taxonomically investigated, these species were categorized into 2 phyla, 4 classes, 6 orders, and 12 families (one of them with an uncertain position). Moreover, the genus *Aspergillus* exhibited 16 species (39.02%). Notably, site no. 6 showed the highest Margalef species richness index at 10.87 followed by site no. 4, while the Shannon diversity index (H) of the recovered taxa was 2.20. Based on the frequency of occurrence, *Aspergillus flavus* recorded the highest percentage (65.56%) followed by *A*. *niger* (50%) and *Penicillium chrysogenum* (40%). Genus *Aspergillus* was recorded in 75 samples (88.33%), while *Penicillium* appeared only in 43 samples, accounting for 47.77% out of 90 samples. The High-performance liquid chromatography (HPLC) analysis showed that aflatoxin B1 (AFB1) was recorded in two animal feedstuff samples at a ratio of 0.851 and 1.363 µg/kg, While AFB2 was discovered in only one animal feedstuff sample at a ratio of 0.479 g/kg. The aflatoxins levels in the positive samples (AFB1 and AFB2) Beef cattle sample components were below the permissible limit for animal feedstuff which is (20 g/kg). Although aflatoxins were found in certain samples, the amounts were much below the maximum residue limits (MRLs) defined by the international authorities or Egyptian guidelines. toxigenic fungi found in contaminated animal feed samples pose a major threat to animal and poultry health, productivity, and even human health. Therefore, periodic monitoring is an excellent way to keep track of their existence and mitigate their hazards.

## 1. Introduction

The variety of fungi in terms of biology and ecology is astounding. Their nutrient intake comes from externally via three primary strategies: saprobic, parasitic, and mutualistic [1]. Species among the kingdom Fungi (including fossil fungi) are categorized into nineteen phyla of fungi. These are Ascomycota, Aphelidiomycota, Basidiobolomyco-ta, Basidiomycota, Caulochytriomycota, Calcarisporiellomycota, Chytridiomycota, Entorrhizomycota, Entomophthoromycota, Glomeromycota, Kickxellomycota, Mortierellomycota, Monoblepharomycota, Mucoromycota, Neocal-limastigomycota, Rozellomycota, Olpidiomycota, and Zoopagomycota [2]. The enzymatic breakdown of organic materials like cellulose, sugars, chitin, lignin, and keratin is how saprobic patterns get their own energy. The parasitic patterns obtain the same material directly from their residing hosts, regardless of whether animals or plants and the latter are eventually killed or destroyed [3].

For better or for worse, fungi have an impact on every aspect of life. These microorganisms have a wide range of beneficial and harmful effects. A serious plant disease is caused by them, which is detrimental to agriculture. Mycotoxins are also deleterious to food, timber, textiles, seeds, grains, and a wide range of many other materials that are either preserved or produced. As a final ramification, they can cause both superficial and deep mycotic infections in humans and animals, which are risky to both [4,5].

Abdel-Azeem documented 2281 fungi taxa from 755 taxonomic groups in 2010, including 57 myxomycete species, by filtering available sources of information [1]. Traditionally, the toxigenic fungi that contaminate grains have been commonly classified to two sets: “field” fungi that attack seed crops (e.g., *Fusarium*, *Cladosporium*, *Alternaria* spp.), which apparently acquire access to seeds in the course of plant development, and there are “storage” fungi (e.g., *Penicillium* spp., *Aspergillus*), that proliferate through storage [6]. Presently, this classification is not fully in accordance to Miller (1995) [7], four toxigenic fungi can be illustrious as follows: (1) plant pathogens, such as *Alternaria alternata and Fusarium graminearum*; (2) fungi that thrive and generate mycotoxins on cellular senescence or stressed plants, including *A.* *flavus*; (3) Fungi that invade the crop early and make the feedstock more vulnerable to contamination after harvesting, e.g., *A*. *flavus*; and (4) Fungi existed in decaying plant or soil debris that arise on emerging kernels in the field and thrive in storage if conditions allow, e.g., *A. ochraceus* and *P. verrucosum*.

*Aspergillus* spp. have been identified as plant diseases, and aflatoxin-infected crops have been reintroduced to agricultural soils on occasion. This practice might be problematic as both *A. parasiticus* and *A. flavus* can infect crops before harvesting [8].

Aflatoxin has been found in apparently healthy, undamaged seeds, suggesting that the toxin may be delivered from contaminated soil to the fruit [9]. Aflatoxin B1 (AFB1) is capable of moving from the roots to the leaves and stems. When the soil microbiota does not swiftly decompose the aflatoxin present in the plowed under grains and stover, the roots of the next year’s crop seedlings may absorb the aflatoxins to both the stems and leaves [10]. This might be harmful to both the plant’s survival and prosperity and the consumer’s health [11].

The quantity of ochratoxin A (OTA) that aggregates in feed and its dispersion in various farm animals were assessed and compared, with a focus on the oral rout administration of OTA-contaminated meals and the subsequent tendency in an animal’s vital organs. The research stated that biocontrol, physical, and chemical strategies to mycotoxin purification in feed are employed all over the globe, but OTA constituted a severe health threat to farm animals [12]. OTA is thought to be an active carcinogen that reduces animal productivity and may be found in meat and meat products [13].

Contamination of animal feedstuff samples and the raw ingredients with filamentous fungi and mycotoxins can happen both before and after harvesting crops [5]. *Aspergillus*, *Penicillium*, *Rhizopus*, and *Cladosporium* are considered the chief contaminants of storage fungi [14]. The main toxigenic species that produce aflatoxins and other toxins are *Aspergillus* species, such as *Aspergillus flavus*, *A*. *parasiticus*, *A*. *nomius*, *A*. *tamarii*, and *A*. *pseudotamarii*. Penicillium and Fusarium are also toxin producers [10,15].

Veterinary diets may comprise cereal grains, primarily maize and/or wheat, and are intended for sick animals. These are frequently infected by *Aspergillus* [10] and *Fusarium* pathogens, which can create a variety of toxic mycotoxins [16].

Corn silage has been demonstrated to alter the degree of contamination of feed delivered to livestock in investigations on the fungal diseases of dairy cattle feed. An increased understanding in this area will aid in elucidating the role of this microbiota in the synthesis and/or degradation of mycotoxins found in silage. Although they are opportunist pathogens, some of these fungi are epidemiologically important and provide a high danger of exposure to field workers who handle them carelessly [17].

The carcinogenic effect of many mycotoxins is well documented for different animal species and humans. Several features in animals, such as age, sex, stress, species, and health status, and various disorders and diseases have been observed, including feed rejection, vomiting, decreased egg and milk production, weakened propagative function, neurotoxicosis, nephrotoxicosis, hepatotoxicosis, cancer, abortions, embryotoxicity, and death [18]. Because various types of cattle foods provide a significant source of various mycotoxins, when animals are fed a diet contaminated with a certain mycotoxin, this may cause a “carry-over” of mycotoxin into their products, which are for consumer consumption [19]. Among the recognized mycotoxins, aflatoxins are considered the most harmful and have been surveyed in several countries worldwide [15,20].

The present study aimed to assess the nutritional and hygienic quality and the incidence of selected fungi groups and mycotoxins in animal feeds in different products not previously studied in Egypt. Ninety samples were collected from five governorates and many subregions, particularly from Cairo (the largest producing area in Egypt). The study involved the enumeration, identification, and biodiversity of mold genera and species, the assessment of the mycotoxins natural levels, such as OTA and aflatoxin, and determination of the suitability of using feeders, and some solutions for deactivating mycotoxins in the feed of animals in Egypt.

## 2. Materials and Methods

### 2.1. Sampling

Ninety samples of various types of animal feedstuff samples that included different components were collected across five governorates in Egypt, as shown in Table 1. The samples were collected in sterile polyethene bags, closed with rubber bands, and transferred directly to the laboratory for further processing.

### 2.2. Isolation and Identification

The technique of dilution plate [21] was adopted to obtain a reasonable amount of fungal diversity. Dichloran Rose Bengal Chloramphenicol Agar (DRBC) supplemented with Rose Bengal (1/15,000) and chloramphenicol (50 ppm), for the suppression of bacterial growth [22], was used as the isolation medium. The taxonomic identification of the isolated fungi utilized a phenotypic method down to the species level on standard media mainly according to the subsequent identification keys: *Penicillium* [23]; *Aspergillus* [24,25,26]; dematiaceous hyphomycetes [27,28]; *Fusarium* [28], miscellaneous fungi [29,30]; and ascomycetes [30]. Recovered taxa were deposited in Suez Canal University Fungarium (http://ccinfo.wdcm.org/collection/by_id/1180, accessed on 1 February 2022). The names of the fungal taxa were abbreviated according to Kirk and Ansell [31]. The presented systematic arrangement is according to the recently reported classification system presented in the 10th edition of Ainsworth and Bisby’s Dictionary of Fungi [32]. The authorities, name corrections, and taxonomic assignments of all the taxa reported in the present study were assessed toward the Index Fungorum database (www.indexfungorum.org, accessed on 1 December 2021).

### 2.3. Equipment and Chemicals

In the present study, the standard and blank aflatoxins B1 and B2 were procured from Sigma Aldrich, Steinheim, Germany, while the Easi-Extract aflatoxin immune-affinity columns (IAC) (OchraTest) were from VICAM, Watertown, MA, USA. The HPLC- solvents grade, including acetonitrile, methanol, and acetone, were obtained from Merck, Darmstadt, Germany. Deionized water and the chemicals and reagents were of the analytical grade. High-performance liquid chromatography (HPLC), Agilent 1100 HPLC system, Agilent Technologies, Waldbronn, Germany, equipped with a quaternary pump model G 1311A, UV detector (Model G 1314A, Agilent, Schönwalde-Glien, Germany) set at a wavelength of 254 nm, with an autosampler (model G1329A VP-ODS, Agilent, Schönwalde-Glien, Germany) and Shim pack (150 × 4.6 mm) column (Shimadzu, Kyoto, Japan) was implemented for aflatoxin determination. The data were integrated and recorded using the Chemstation Software program.

The reagents used are all HPLC standards. Sigma (St. Louis, MO, USA) provided the ochratoxin A and aflatoxin standards, anhydrous sodium sulphate, acetic acid, tetrahydrofuran and diatomaceous earth, whereas hexane, chloroform, acetone, methanol, toluene, formic acid, ethyl acetate, Tris-hydrochloric acid, orthophosphoric acid, and HPLC grade water were acquired from Merck Inc.(Merck, Darmstadt, Germany).

The solid standard of AFG1, AFG2, AFB1, AFB2, and OCA were dissolved in benzene:acetonitrile mixture (98:2, *v*/*v*). The actual concentration was determined in accordance with AOAC utilizing the Shimadzu UV-1601 PC spectrophotometer (Shimadzu Scientific Instruments, Japan) [33]. Standard solutions of 9.855 ng mL^−1^ concentration were also prepared in benzene:acetonitrile (98:2, *v*/*v*) and were utilized for plotting the calibration curve in the range 0.1–9.8 ng/mL. The standard solutions were kept at −18 °C in amber-colored vials.

### 2.4. Quantitative Determination of Aflatoxins

Aflatoxins were recovered by treating 50 g of the samples for 10 minutes with acetone (100 mL) and water (100 mL). 10 g of diatomaceous earth were added to the reaction mixture and gently stirred for 5 minutes prior to filtration through swift filtering filter paper (Whatman No. 1). Precisely, An Erlenmeyer volumetric flask (500 mL) was filled with 100 L of the filtrate and mixed with 5% NaCl (50 mL), and Hexane (50 mL), and the mixture was agitated at 2400 rpm for 5 min by a mechanical shaker. (IKA, GmbH, Breisgau, Germany). The hexane layer was removed. Following that, 5% NaCl (50 mL) and chloroform (150 mL) were dissolved in aqueous layer and gently agitated for 5 min. The chloroform layer was extracted three times, dehydrated over Na_2_SO_4_, and concentrated under reduced pressure. 1 mL chloroform was used to re-dissolve the leftovers [34]. An aqueous acetic acid (2 mL, 0.5%) was used to precondition the column, followed by the extracts (1 mL) and acetic acid (4 mL, 0.5%) injected over C18 column. The column was then treated with aqueous acetic acid (0.5 mL, 0.5%) and 20% tetrahydrofuran (THF) to be cleaned. The column was then run through with 2 mL of hexane and dried with nitrogen. The column was rinsed with THF in hexane (3 mL, 25%) and dehydrated for 1 minute using nitrogen. The aflatoxins were eluted using THF in methylene chloride (2 mL, 1%), followed by dehydration over a nitrogen pressure. Prior HPLC injection, the powdered aflatoxins were recovered in methanol (0.5 mL). Each aflatoxin was assessed using HPLC at wavelengths of 365 and 440 nm for excitation and emission, respectively. The mobile phase, composed of toluene, ethyl acetate, methanol and formic acid (90:5:2.5:2.5, *v*/*v*), was injected with a 1.0 mL/min steady flow. The sample was then put into the HPLC system at 24 °C in order to acquire the best possible resolution of the aflatoxins. Many blanks (methanol alone) and aflatoxin standard solutions were already injected tandemly. The evaluation of the samples was carried out in triplicates, and the sample was considered to be positive for aflatoxin if the retention duration and peak of the sample corresponded to that of the standard. The Agilent ChemStation Software System was used to determine the amount of each aflatoxin that was present in the samples that were analyzed.

### 2.5. Quantitative Determination of Ochratoxin A

The sample extraction was performed according to Toscani et al. [35], where an aliquot of the prepared sample (10 g) and a mixture of chloroform in orthophosphoric acid (100 mL, 85%, 100:4, *v*/*v*) were mixed for 2 min in a blender. After being mixed, the ingredients were filtered using Whatman No. 3 filter paper, and then the resulting filtrate (60 mL) were transferred to a separating funnel and extracted (2× 5 mL) with a buffer comprising 0.2 M Tris-HCl:acetonitrile 90:10 (*v*/*v*). The aqueous layer was carefully combined and probably mixed. Precisely, the aliquot (50 mL) was injected for cleanup to Agilent ZORBAX C18 column (3 μm, 2.1 × 250 mm). The column was rinsed with H_2_O and drying with air. Using a vacuum manifold, ochratoxin A was eluted with methanol (2 mL). The methanol was removed with nitrogen stream and the residue was redissolved in the mobile phase (acetonitrile:H_2_O:glacial acetic acid 49.5:49.5:1.0), and injected into the HPLC system. The ochratoxin A was assessed at wavelengths of 380 and 440 nm for excitation and emission, accordingly. The mobile phase applied consist of H_2_O:acetonitrile:glacial acetic acid 49.5:49.5:1.0; which was injected with a 1 mL/min steady flow. Typically, the same techniques and steps were used for the determination of the aflatoxins and applied to estimate ochratoxin A with the Agilent ChemStation Software System.

### 2.6. Detection of Mycotoxins in Fungal Fermentation

The fungal cultures were grown in 50 mL potato dextrose broth (PDB) and were incubated for 10 days at 28 °C [36] followed by the extraction of mycotoxins according to a previous study [37] where the broth cultures were homogenized and extracted with 100 mL of chloroform by shaking at 150 rpm for 24 h. The extraction was repeated two times with the same volume of chloroform. Following the extraction, the mixture was filtered, and the chloroform part was separated from the aqueous part using separating funnels. Furthermore, the chloroform extracts were dried over anhydrous sodium sulfate in a steam bath. Thin-layer chromatography (TLC) was used for the detection of the mycotoxins [38] using chloroform:methanol (96:4 *v*/*v*) as a solvent system. The TLC plates were visualized under a short and long UV light. The identified mycotoxins were compared with the appropriate reference standards, as previously described [36,39,40].

### 2.7. Statistical Analyses

The data were collected, checked, revised, and presented in the tables and figures using Microsoft Excel 2016 (16.0 version, Microsoft, Redmond, WA, USA). Normality was checked using Kolmogorov–Smirnov to detect whether the data were parametric or nonparametric at the 0.05 level. Differences between taxonomic groups were checked using the chi-square and Kruskal–Wallis test statistics. The mycotoxins assays from different fungal isolations from various feeding sources were evaluated using a two-way analysis of variance. Fungal diversity was studied using the Margalef and Shannon diversity indices according to Muthukrishnan (2012) [41]. The statistical analyses were carried out using IBM-SPSS version 28.0 for Mac OS [42,43,44].

## 3. Results and Discussion

Mycological examination of 90 animal feedstuff samples revealed that 79 (87.77%) samples were contaminated with different fungal species (1.1 × 10^5^ CFU/g) (Table 2). The fungal load among the samples varied from 17 to 3.3 × 10^3^ CFU/g, with sample no. 4 being heavily contaminated, which was obtained from rice hulls collected from Alexandria. Other samples of corn bran, yellow corn, broad bean hulls, wheat bran, sorghum grains, soybean hulls, beef cattle feed, and date waste from Alexandria, Beheira, New Valley, and Cairo were also contaminated with relatively high numbers of fungal units.

Taxonomically, the identified taxa were classified to two phyla with four classes, six orders, and eleven families. The order Eurotiales showed the highest species range (28 species) followed by Hypocreales (5 species), Sordariales, and Mucorales (3 species each), while the residual showed the lowest range (1 species). The family Aspergillaceae contributed maximally (28 fungal species out of 41) followed by Chaetomiaceae (3 species), and the remaining families were characterized only by one or two species. On the higher taxa level, Zygomycota was characterized by three species (7.31% of the total species number), teleomorphic Ascomycota had 8 species (19.51%), and anamorphic Ascomycota had 30 species (73.17%). The species identified belonged to 18 genera (Table 2).

*Aspergillus* was the main genus from the fungal contaminated samples, with a total count of about 39.02% of the total fungal population. Sixteen species of *Aspergillus* were identified, of which *A. flavus* and *A. niger* were the most commonly colonizing fungi at 65.56% and 50%, respectively (Table 2).

The genus *Aspergillus* was recorded in 75 samples (88.33%), while *Penicillium* appeared in only 43 samples, accounting for 47.77% of the total samples. The remaining species belonged to the genera *Acremonium*, *Fusarium*, *Botryotrichum*, *Chaetomium*, *Eupenicillium*, *Trichocladium*, *Lichtheimia*, *Monascus*, *Mucor*, *Paecilomyces*, and *Talaromyces* (Table 2).

A statistically significant difference among species (*p* < 0.001 ***), TC (*p* < 0.001 ***), TC% (*p* < 0.001 ***), F% (*p* < 0.001 ***), phyla (*p* < 0.001 ***), classes (*p* < 0.001 ***), orders (*p* < 0.001 ***), and families (*p* < 0.001 ***) was revealed by the chi-square test (Table 2 and Table 3).

Concerning the biodiversity among the sites, site 6 had the highest Margalef species richness index at 10.87, followed by site 4, and the Shannon diversity index (H) of the identified taxa was 2.20. In terms of the frequency of occurrence, *Aspergillus flavus* had the highest percentage (65.56 percent), followed by Aspergillus niger (50 percent), and *Penicillium chrysogenum* (50 percent) (40 percent). The genus *Aspergillus* was found in 75 samples (88.33 percent), whereas *Penicillium* was found in only 43 (47.77 percent) of the 90 samples.

**Figure 1:**
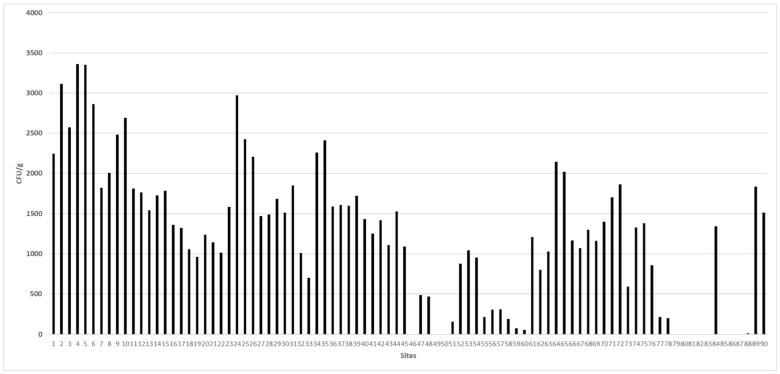
The fungal load in all the collected samples across various sites in Egypt.

The present study analyzed 13 animal feedstuff samples for mycotoxin contamination due to fungal contamination. The lowest and highest fungal yields observed were 2.2 × 10^3^ CFU/g and 2.9 × 10^3^ CFU/g, respectively, as depicted (Table 4, Figure 1).

*Aspergillus flavus* and *A*. *niger* were the main species in the studied samples. Samples no. 24 and 26 were contaminated with aflatoxin B1 (0.851 and 1.363 µg/kg, respectively) (Figure S1). Aflatoxin B2, at a ratio of 0.479 µg/kg, was recorded in sample 26. Notably, the current investigation found reduced amounts of aflatoxins. (AFB1 and AFB2) in the positive samples (Figures S2 and S3) in contrast to the permissible ranges for beef cattle animal feedstuff ingredients (20 µg/kg) as reported by good manufacturing practices (GMP) [45].

According to Table 5, various mycotoxins were recorded from the different feeding sources. The mycotoxins assayed included aflatoxins (B1, B2) and ochratoxin A, which were present at a statistically significant level (*p* = 0.004 **), as revealed by the two-way analysis of variance. The recorded mycotoxins were assayed from different sources, and fungi were statistically significant (*p* = 0.007 ***), as revealed by the two-way analysis of variance. The highest average level of aflatoxins was 100 in the poultry feed, broad bean hulls, and yellow corn from the fungal isolate *A*. *flavus*. In addition, a bean hull revealed aflatoxin B2 at 75 (SD = 50.0) from *A. flavus*.

Only six *A. flavus* isolates were capable of producing aflatoxins B1 and B2 at estimated levels of 100 to 200 g/L when 30 fungal isolates representing *A. flavus* [17], *A. niger* [7], *A. ochraceus* [2], and *A. parasiticus* [4] were tested for their abilities to produce mycotoxins. These isolates were obtained from poultry feeds, bean hulls, rice hulls, and yellow corn (Table 4). Mycotoxins were not produced in detectable amounts by the remaining *Aspergillus* isolates.

According to various studies conducted by numerous investigators [33,34], the quality of animal feed is a critical requirement for achieving the best animal product yield as well as the preservation of the animals’ health conditions, particularly in intensive industrial production. A periodic analysis was required for the fungal assessment and evaluation of mycotoxin contamination levels, as well as the 41 fungal species from the 18 fungal genera obtained from the various animal feedstuff samples (Table 2). The final genus isolated from mycologically pure materials was *Aspergillus,* it had such a high total count that it equaled the whole fungal biomass (39.02 percent).

Consistent with the current study, a study in Serbia [46] showed the incidence of fungi in 297 samples of animal feedstuff (maize, ground maize, sunflower, soybean meal, and finished mixture). The total fungal count was 1–9 × 10^4^ CFU/g in most of the examined animal feed samples, with the most prevalent fungi being *Fusarium* (100% of samples) followed by *Aspergillus* (87.10%), *Rhizopus* (83.87%), *Penicillium* (70.32%), and Mucor (33.55%). Similarly, in Argentina, [47], mycobiota was assessed from corn silage, corn grains, peanut shells, soybean, wheat bran, and alfalfa hay and exhibited a fungal load from 0–2.10 × 10^8^ CFU/g with the presence of aflatoxin B1 (AFB1), fumonisin B1 (FB1), deoxynivalenol (DON), and zearalenone (ZEA) levels in total mixed rations (TMRs) of cattle feed. The dominant genera revealed were *Aspergillus* spp. (60%) and *Fusarium* spp. (66.7%), among which *Aspergillus fumigatus* was predominantly present along with other fungal species, such as *A*. *niger*, *A*. *flavus*, *Fusarium proliferatum*, *F*. *subglutinans, F*. *verticillioides, Penicillium brevicompactum, P*. *griseofulvin, P*. *roqueforti,* and *P*. *crustosum,* as well as representative species of Alternaria, *Cladosporium,* and *Geotrichum*.

The current investigation evaluated 30 fungal strains representing *A. flavus* (17 strains), *A. niger* [7], *A. ochraceus* [2], and *A. parasiticus* [4] for their ability to create mycotoxins. Only six strains of A. flavus were capable of producing aflatoxins B1 and B2 at estimated concentrations of 100–200 g/L. These isolates were from poultry feeds, bean hulls, rice hulls, and yellow corn (Table 5). The remaining Aspergillus strains produced no detectable mycotoxins.

Similar to this investigation, 186 cow feedstuff samples were tested in Iran from traditional and industrial dairy farms in the Hamadan district [45]. The most prevalent fungi were *Mucor* species (3.4%), *Rhizopus* (3.9%), *Alternaria* (4.3%), *Cladosporium* (9.1%), *Fusarium* (17.5%), *Penicillium* (23.7%), and *Aspergillus* species (37.3%). *A. clavatus*, *A. fumigatus*, *A. niger*, *A. flavus*, *A. parasiticus*, *A. nidulans*, *A. terreus*, *A. ochraceus*, and *A. ustus* were all *Aspergillus* species. Additionally, the authors observed that concentrate feed and wheat bran were the most contaminated feedstuffs, with the mean colony counts for *A. flavus* and *A. parasiticus* at 7.25 102 and 7.50 102 CFU/g, respectively. These fungi were likewise isolated from animal feedstuff samples evaluated in our investigation, except for *Alternaria* and *Cladosporium*. Other data from Iran [36,46] indicated fungal contamination in 31.3% of chicken feed samples. Similarly, 51 samples of animal feed were examined in India to determine the presence of toxigenic fungus [47]. This analysis discovered that the *Aspergillus* species was the most prevalent fungi found in 83% of samples. *A. flavus* was the most prevalent *Aspergillus* species, followed by *A. niger* and *A. fumigatus*. *Penicilium*, followed by *Fusarium*, colonized the feed samples after *Aspergillus*. In Saudi Arabia [48], 20 samples of soybean meal used in animal feed were evaluated. Al-Seeni stated that the overall fungal counts varied between 3.9 × 10^4^ and 10^5^ CFU/g. The frequently isolated fungi included *Aspergillus*, *Fusarium*, and *Penicillium*. Mycotoxins identified in animal feedstuff samples, namely, soybean, ranged between 1 and 14.8 parts per billion (ppb) for aflatoxins and 1 to 14 parts per billion (ppb) for ochratoxin A. Although the majority of the mycobiota recovered by [38] are identical to those obtained in this study, the current analysis showed that the number of mycotoxins in the Saudi Arabian samples was significantly greater. Mycotoxins’ potential risks can be rapidly mitigated by inspecting plant material for fungal contamination, harvesting, enhancing cultivation, and storage methods, abolishing or diluting toxins from contaminated feeds or foods, and utilizing adsorbents to diminish the toxins’ bioavailability in animal digestive tracts [48].

## 4. Conclusions

This study showed that animal feed collected from various locations in Egypt, as well as their ingredients, are frequently contaminated with mycotoxigenic fungi and mycotoxins where the main reservoirs for mycotoxins in animal feed are ingredients, particularly rice hulls, corn bran, yellow corn, and broad bean hulls. Many health studies have been performed in the last three decades to explore the hazardous effects of the presence of mycotoxins in animal feeds and human food. The general teratogenicity, toxicity, and carcinogenicity of these molecules constitute a risk to human and animal health. Based on our results, this situation demands immediate necessary control measures. Adequate postharvest drying should be performed, and proper storage conditions should be maintained. Egyptian farmers should be educated and sensitized about proper storage facilities, duration, and conditions for feed and ingredients. To reduce mycotoxin contamination in feed ingredients and finished animal feed, antifungal agents should be used. More research is needed to investigate the synergistic effects of mycotoxins when diets contain more than one mycotoxin. Animal feed is such a critical component of the food chain and must require the quality assurance of rigorous food safety systems. Finally, To promote consumer health, strong restrictions and monitoring systems for testing food and feed for aflatoxin and ochratoxin contamination are strongly recommended. We assume that periodic assessing and the utilize of critical control points (HACCP) and hazard analysis for the control and prevention of mycotoxins in the animal feed industry are required to ensure the feed hygienic quality, productivity and animal health, and prevent human foodborne diseases.

## Figures and Tables

**Table 1:** Types of animal feedstuff samples ingredients collected from different localities in Egypt.

Sites No.	Ingredient of Animal Feedstuff Samples	Locality	Governorate
1–6	Rice hulls	Amereyah	Alexandria
7–11	Corn bran	Amereyah	Alexandria
12–17	Yellow Corn	Amereyah	Alexandria
18–33	Broad bean hulls	Rasheed	Beheira
34–39	Beef cattle feed	Kharga	New Valley
40	Alfalfa hay	Kharga	New Valley
41, 42, 45	Date waste	Kharga	New Valley
43	Soy bean	Kharga	New Valley
44	Wheat bran	Kharga	New Valley
46	Broiler concentrate	Kharga	New Valley
47	Yellow corn	Kharga	New Valley
48	Date waste	Kharga	New Valley
49	Broiler poultry feed (19% protein)	Semouha	Alexandria
50	Broiler poultry feed (23% protein)	Semouha	Alexandria
51, 52	Rabbit feed	Cairo	Cairo
53	Rice hulls	Cairo	Cairo
54	Yellow corn	Cairo	Cairo
55	Broiler feed	Cairo	Cairo
56	Wheat bran	Cairo	Cairo
57	Yellow corn	Cairo	Cairo
58	Wild barley	Cairo	Cairo
59	Yellow Corn	Cairo	Cairo
60	Soybean	Cairo	Cairo
61	Complete feedstuff	Cairo	Cairo
62	Bean hulls	Cairo	Cairo
63, 64	Sorghum grains	Cairo	Cairo
65	Soybean hulls	Cairo	Cairo
66	Wheat grains	Cairo	Cairo
67, 68	Broiler feed	Cairo	Cairo
69	Fine ground corn	Assiut	Assiut
70	Coarse ground corn	Assiut	Assiut
71	White corn	Assiut	Assiut
72	Bean hulls	Assiut	Assiut
73	Wheat bran	Assiut	Assiut
74–78	Layer strain poultry feed (17% protein)	Semouha	Alexandria
79	Magnesium sulphate	Assiut	Assiut
80	Dicalcium phosphate	Assiut	Assiut
81	Methionine	Assiut	Assiut
82	Lime	Assiut	Assiut
83	Premix for layer strains	Assiut	Assiut
84	Salts for poultry	Assiut	Assiut
85	Vitamins A, D	Assiut	Assiut
86	Lysine	Assiut	Assiut
87	Salts for cattle	Assiut	Assiut
88	Premix for beef cattle feed	Assiut	Assiut
89	Beef cattle feed	Assiut	Assiut
90	Poultry feed (17% protein)	Assiut	Assiut

**Table 2:** Species list recorded during the study.

Fungal Genera and Species	TC	% TC	Freq.	% F	Phylum	Class	Order	Family
*Acremonium roseolum* (G. Smith) W. Gams	40	0.036	1	1.1	Ascomycota	Sordariomycetes	Hypocreales	Incertae sedis
*Aspergillus* (Total)		0.000		0.0	Ascomycota	Eurotiomycetes	Eurotiales	Aspergillaceae
*A. egyptiacus* Moubasher & Moustafa	40	0.036	1	1.1	Ascomycota	Eurotiomycetes	Eurotiales	Aspergillaceae
*A. amstelodami* (L. Mangin) Thom & Church	6320	5.624	15	16.7	Ascomycota	Eurotiomycetes	Eurotiales	Aspergillaceae
*A. candidus* Link	10,380	9.238	23	25.6	Ascomycota	Eurotiomycetes	Eurotiales	Aspergillaceae
*A. clavatus* Desmazieres	40	0.036	1	1.1	Ascomycota	Eurotiomycetes	Eurotiales	Aspergillaceae
*A. flavus* Link	18,920	16.838	59	65.6	Ascomycota	Eurotiomycetes	Eurotiales	Aspergillaceae
*A. flavipes* (Bain. & Sart.) Thom & Church	920	0.819	11	12.2	Ascomycota	Eurotiomycetes	Eurotiales	Aspergillaceae
*A. fumigatus* Fresenius	1600	1.424	9	10.0	Ascomycota	Eurotiomycetes	Eurotiales	Aspergillaceae
*A. niger* van Tieghem	41,251	36.711	45	50.0	Ascomycota	Eurotiomycetes	Eurotiales	Aspergillaceae
*A. nidulans* (Eidam) G. Winter	200	0.178	5	5.6	Ascomycota	Eurotiomycetes	Eurotiales	Aspergillaceae
*A. ochraceus* Wilhelm	440	0.392	5	5.6	Ascomycota	Eurotiomycetes	Eurotiales	Aspergillaceae
*A. parasiticus* Speare	920	0.819	10	11.1	Ascomycota	Eurotiomycetes	Eurotiales	Aspergillaceae
*A. ruber* (Jos. König, Spieck. & W. Bremer) Thom & Church	4000	3.560	25	27.8	Ascomycota	Eurotiomycetes	Eurotiales	Aspergillaceae
*A. sydowii* (Bainier & Sartory) Thom & Church	4520	4.023	20	22.2	Ascomycota	Eurotiomycetes	Eurotiales	Aspergillaceae
*A. terreus* Thom	3680	3.275	20	22.2	Ascomycota	Eurotiomycetes	Eurotiales	Aspergillaceae
*A. versicolor* (Vuillemin) Tiraboschi	120	0.107	1	1.1	Ascomycota	Eurotiomycetes	Eurotiales	Aspergillaceae
*A. ustus* (Bainier) Thom & Church	40	0.036	1	1.1	Ascomycota	Eurotiomycetes	Eurotiales	Aspergillaceae
*Botryotrichum atrogriseum* J.F.H. Beyma	40	0.036	1	1.1	Ascomycota	Sordariomycetes	Sordariales	Chaetomiaceae
*Chaetomium globosum* Kunze	120	0.107	2	2.2	Ascomycota	Sordariomycetes	Sordariales	Chaetomiaceae
*Eupenicillium inusitatum* D.B. Scott	800	0.712	4	4.4	Ascomycota	Eurotiomycetes	Eurotiales	Aspergillaceae
*Fusarium* (Total)	0	0.000	2	2.2	Ascomycota	Sordariomycetes	Hypocreales	Nectriaceae
*F. incarnatum* (Desm.) Sacc.	40	0.036	1	1.1	Ascomycota	Sordariomycetes	Hypocreales	Nectriaceae
*F. solani* (Martius) Saccardo	40	0.036	1	1.1	Ascomycota	Sordariomycetes	Hypocreales	Nectriaceae
*Trichocladium griseum* (Traaen) X. Wei Wang & Houbraken	200	0.178	3	3.3	Ascomycota	Sordariomycetes	Sordariales	Chaetomiaceae
*Lichtheimia corymbifera* (Cohn) Vuill.	360	0.320	5	5.6	Zygomycota	Mucoromycetes	Mucorales	Cunningham-ellaceae
*Monascus purpureus* Went	320	0.285	5	5.6	Ascomycota	Eurotiomycetes	Eurotiales	Monascaceae
*Mucor* hiemalis Wehmer	40	0.036	1	1.1	Zygomycota	Mucoromycetes	Mucorales	Mucoraceae
*Paecilomyces variotii* Bainier	480	0.427	5	5.6	Ascomycota	Eurotiomycetes	Eurotiales	Aspergillaceae
*Penicillium* (Total)	0	0.000	45	50.0	Ascomycota	Eurotiomycetes	Eurotiales	Aspergillaceae
*P. aurantiogriseum* Dierckx	200	0.178	4	4.4	Ascomycota	Eurotiomycetes	Eurotiales	Aspergillaceae
*P. chrysogenum* Thom	10,420	9.273	36	40.0	Ascomycota	Eurotiomycetes	Eurotiales	Aspergillaceae
*P. citrinum* Thom	560	0.498	6	6.7	Ascomycota	Eurotiomycetes	Eurotiales	Aspergillaceae
*P. solitum* Westling	80	0.071	2	2.2	Ascomycota	Eurotiomycetes	Eurotiales	Aspergillaceae
*Talaromyces duclauxii* (Delacr.) Samson, N. Yilmaz, Frisvad & Seifert	680	0.605	10	11.1	Ascomycota	Eurotiomycetes	Eurotiales	Aspergillaceae
*P. glabrum* (Wehmer) Westling	160	0.142	2	2.2	Ascomycota	Eurotiomycetes	Eurotiales	Aspergillaceae
*T. islandicus* (Sopp) Samson, N. Yilmaz, Frisvad & Seifert	160	0.142	2	2.2	Ascomycota	Eurotiomycetes	Eurotiales	Aspergillaceae
*T. pinophilus* (Hedgc.) Samson, N. Yilmaz, Frisvad & Seifert	3160	2.812	30	33.3	Ascomycota	Eurotiomycetes	Eurotiales	Aspergillaceae
*T. samson* N. Yilmaz, Houbraken, Spierenb., Seifert, Peterson, Varga & Frisvad [as ‘purpurogenus’]	320	0.285	5	5.6	Ascomycota	Eurotiomycetes	Eurotiales	Aspergillaceae
*Didymella glomerata* (Corda) Qian Chen & L. Cai	40	0.036	1	1.1	Ascomycota	Dothideomycetes	Pleosporomycetidae	Didymellaceae
*Rhizopus arrhizus* A. Fisch.	40	0.036	1	1.1	Zygomycota	Mucoromycetes	Mucorales	Rhizopodaceae
*Microascus brevicaulis* S.P. Abbott	80	0.071	2	2.2	Ascomycota	Sordariomycetes	Microascales	Microascaceae
*Stachybotrys chartarum* (Ehrenberg) Hughes	396	0.352	5	5.6	Ascomycota	Sordariomycetes	Hypocreales	Stachybotryaceae
*Trichoderma harzianum* Rifai	200	0.178	2	2.2	Ascomycota	Sordariomycetes	Hypocreales	Hypocreaceae
Gross Total Count	112,367	100	-					
Chi-square test/Kruskal–Wallis	<0.001 ***	<0.001 ***	<0.001 ***	<0.001 ***	<0.001 ***	<0.001 ***	<0.001 ***	<0.001 ***

*** indicates *p* < 0.001.

**Table 3:** Chi-square test for assessing the difference by species, TC, frequency, and taxonomic groups. (*** indicates *p* < 0.001).

	Chi-Square	df	Sign.
Species	743.4	40	<0.001 ***
TC	544.7	51	<0.001 ***
TC %	310.1	23	<0.001 ***
F %	153.2	15	<0.001 ***
Phylum	352.5	1	<0.001 ***
Class	973.3	3	<0.001 ***
Order	1868.9	6	<0.001 ***
Family	2049.3	6	<0.001 ***

**Table 4:** Mycotoxins detected in animal feedstuff samples.

Sample No.	Fungal Load (CFU/g)	Aflatoxins (µg/kg)				Ochratoxin A
		B1	B2	G1	G2	
1	2.2 × 10^3^	-	-	-	-	-
2	3.1 × 10^3^	-	-	-	-	-
3	2.5 × 10^3^	-	-	-	-	-
4	3.3 × 10^3^	-	-	-	-	-
6	2.8 × 10^3^	-	-	-	-	-
10	2.6 × 10^3^	-	-	-	-	-
17	1.3 × 10^3^	-	-	-	-	-
18	1.0 × 10^3^	-	-	-	-	-
24	2.9 × 10^3^	0.851	-	-	-	-
25	2.4 × 10^3^	-	-	-	-	-
26	2.2 × 10^3^	1.363	0.479	-	-	-
30	1.5 × 10^3^	-	-	-	-	-
32	1.0 × 10^3^	-	-	-	-	-

**Table 5:** Assay of mycotoxins from different fungal isolated from various feeding sources.

All	(ug/Liter) Using TLC													
	Name	Source	Mycotoxins	Source	Fungi	Mycotoxin	N	Mean	SD	SE	CI 95%		Min	Max
											Lower	Upper		
1	*A. flavus*	Rice hulls	Aflatoxins B1, B2	1	1	1	6	0	0	0	0	0	0	0
2		Corn bran		2	1	1	1	0	0	0	0	0	0	0
3		Yellow corn		3	1	1	2	0	0	0	0	0	0	0
4		Broad bean hulls		4	1	1	1	0	0	0	0	0	0	0
5		Poultry feed		5	1	1	1	0	0	0	0	0	0	0
6		Poultry feed	Aflatoxin B1	5	1	2	1	100	0	0	0	0	100	100
7		Broad bean hulls	Aflatoxin B2	5	1	3	1	100	0	0	0	0	100	100
8		Bean hulls	Aflatoxin B1	4	1	2	4	100	81.6	40.8	−30	230	0	200
9		Bean hulls	Aflatoxin B2	4	1	3	4	75	50	25	−4.6	155	0	100
10		Yellow corn	Aflatoxin B1	3	1	2	1	100	0	0	0	0	100	100
11	*A. niger*	Beef cattle feed	Ochratoxin A	6	2	4	5	0	0	0	0	0	0	0
12		Alfalfa hay		7	2	4	1	0	0	0	0	0	0	0
13		Date wastes		1	2	4	1	0	0	0	0	0	0	0
14	*A. ochraceus*	Rice hulls	Ochratoxin A	1	3	4	2	0	0	0	0	0	0	0
15	*A. parasiticus*	Layer strain poultry feed	Aflatoxins	5	4	1	4	0	0	0	0	0	0	0
Source of variation			df		F					Sign.				
Corrected model			14		3.321					0.007 **				
Intercept			1		12.854					0.002 **				
Source			6		0.017					>0.05 ns				
Fungi			2		0					>0.05 ns				
Mycotoxin			2		7.361					0.004 **				
Source * Mycotoxin			3		0.076					>0.05 ns				

** indicates *p* < 0.004, * indicates *p* < 0.007.

## Data Availability

Not applicable.

## Supplementary Materials

The following supporting information can be downloaded at: https://www.mdpi.com/article/10.3390/ijerph19127250/s1, Figure S1. HPLC chromatogram of aflatoxin B1 in the examined feed samples; Figure S2. HPLC chromatogram of aflatoxin B1 in the examined feed samples; Figure S3. HPLC chromatogram of aflatoxin B2 in the examined feed samples.
