# Supplementary material for: Diversity of Toxigenic Fungi in Livestock and Poultry Feedstuffs
Source: Int J Environ Res Public Health. 2022 Jun 13;19(12):7250. doi: 10.3390/ijerph19127250 (PMC9224174; doi:10.3390/ijerph19127250)
Supplement: Supplementary file 1 [file ijerph-19-07250-s001.zip › ijerph-1749867-supplementary.pdf]

Supporting Information

Diversity of toxigenic fungi in livestock and poultry feedstuffs

Eman Khalifa [1]†, Marwa T. Mohesien [2], Monga I. Mossa [3], Magdalena Piekutowska [4], Amnah Mohammed Alsuhaibani [5], Basel A. Abdel-Wahab [6,7], Sotohy Ahmed Sotohy [8] , Soumya Ghosh [9], Yosra A. Helmy [10,11], Mohamed Hussein [12]† and Ahmed M. Abdel-Azeem*†[13]

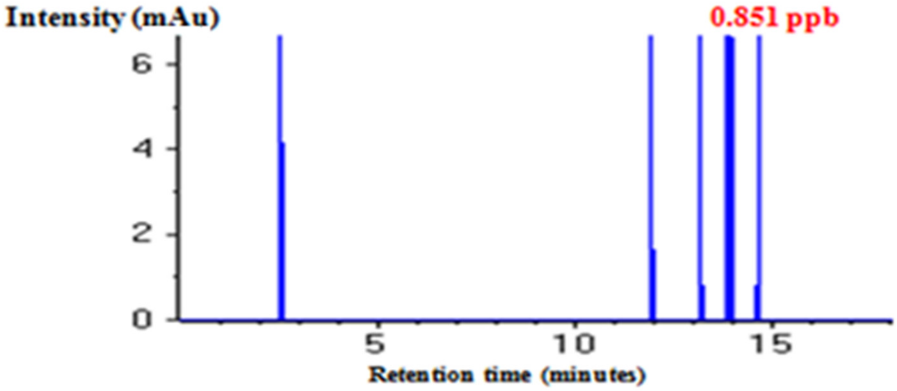

Figure S1. HPLC chromatogram of aflatoxin B1 in the examined feed samples.

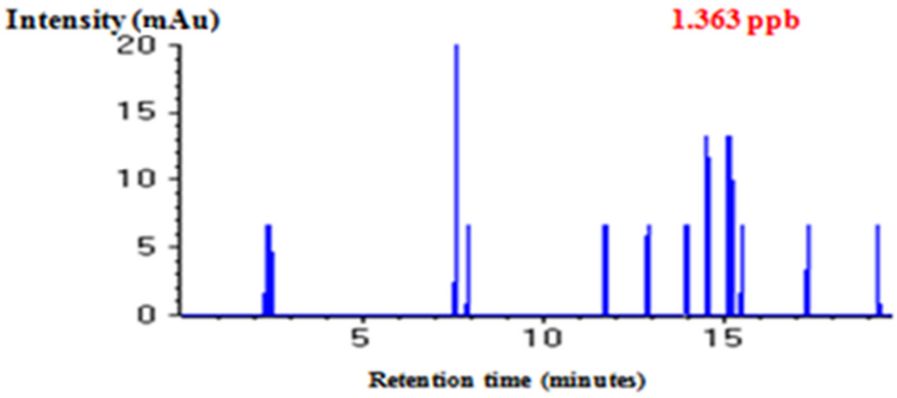

Figure S2. HPLC chromatogram of aflatoxin B1 in the examined feed samples.

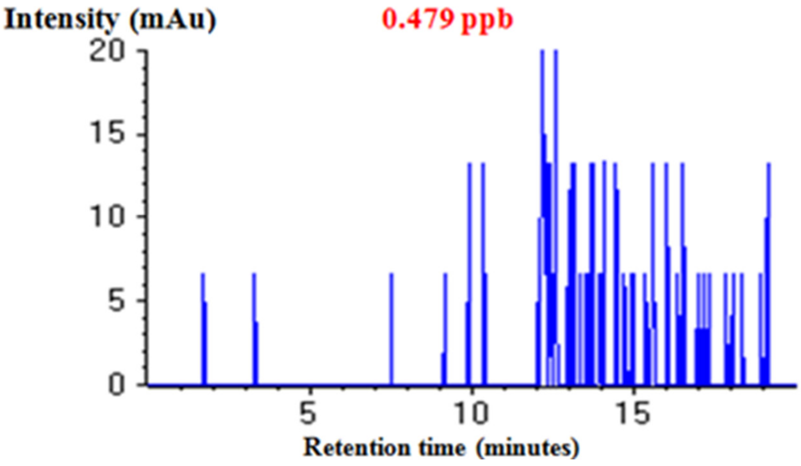

Figure S3. HPLC chromatogram of aflatoxin B2 in the examined feed samples.
